# CEACAM expression in an *in-vitro* prostatitis model

**DOI:** 10.3389/fimmu.2023.1236343

**Published:** 2023-08-25

**Authors:** Irina Kube-Golovin, Mykola Lyndin, Marc Wiesehöfer, Gunther Wennemuth

**Affiliations:** ^1^ University Hospital Essen, Department of Anatomy, Essen, Germany; ^2^ Academic and Research Medical Institute, Department of Pathology, Sumy State University, Sumy, Ukraine

**Keywords:** prostatitis, inflammation, CEACAM, CEACAM1, prostate cancer

## Abstract

**Background:**

Prostatitis is an inflammatory disease of the prostate gland, which affects 2-16% of men worldwide and thought to be a cause for prostate cancer (PCa) development. Carcinoembryogenic antigen-related cell adhesion molecules (CEACAMs) are deregulated in inflammation and in PCa. The role of CEACAMs in prostate inflammation and their possible contribution to the malignant transformation of prostate epithelial cells is still elusive. In this study, we investigated the expression of CEACAMs in an *in-vitro* prostatitis model and their potential role in malignant transformation of prostate epithelial cells.

**Methods:**

Normal prostate epithelial RWPE-1 cells were treated with pro-inflammatory cytokines to achieve an inflammatory state of the cells. The expression of CEACAMs and their related isoforms were analyzed. Additionally, the expression levels of selected CEACAMs were correlated with the expression of malignancy markers and the migratory properties of the cells.

**Results:**

This study demonstrates that the pro-inflammatory cytokines, tumor necrosis factor alpha (TNFα) and interferon-gamma (IFNγ), induce synergistically an up-regulation of CEACAM1 expression in RWPE-1 cells, specifically favoring the CEACAM1-L isoform. Furthermore, overexpressed CEACAM1-L is associated with the deregulated expression of JAK/STAT, NFκB, and epithelial-mesenchymal transition (EMT) genes, as well as an increased cell migration.

**Conclusion:**

We postulate that CEACAM1 isoform CEACAM1-4L may synergistically contribute to inflammation-induced oncogenesis in the prostate.

## 1 Introduction

Prostatitis refers to an inflammation of the prostate gland, which affects 2-16% of men worldwide, with a higher incidence observed in middle-aged individuals (1–5). Prostatitis is clinically heterogeneous and comprises acute bacterial prostatitis, chronic bacterial prostatitis, chronic pelvic pain syndrome, and asymptomatic inflammatory prostatitis, according to the US National Institutes of Health (NIH) (6). The inflammatory response within the prostate can be induced by systemic disseminated or an organ-restricted infection (7). Potential sources of prostatitis include sexually transmitted infections, physical trauma, and chemical exposure induced by urine reflux, diet, estrogens, or a combination of two or more of these factors (8–10).

About 20% of all human cancers are caused by chronic infection or chronic inflammation (11) and evidence arised that prostatitis may lead to PCa initiation and progression to a metastatic disease (8, 12, 13). In particular, acute prostatitis has been correlated with an increased risk of developing PCa (14). However, the underlying mechanisms are not fully understood so far.

CEACAMs belong to the immunoglobulin superfamily (15) and can be expressed in epithelial (16, 17), endothelial (18), and immune cells (19, 20). CEACAMs are engaged in cell-cell communication that influences a variety of signaling events, including those involved in mitogenesis, survival/apoptosis, differentiation, migration, invasion, arrangement of three-dimensional tissue structure, angiogenesis, and modulation of immune responses (21, 22). The most extensively characterized member of the CEACAM family is CEACAM1. It consists of an N-terminal ectodomain (N-domain), extracellular Ig-like domains (AB-domains), a conserved transmembrane domain, and either a short (CEACAM1-S) or long cytoplasmic domain (CEACAM1-L) generated due to the alternative splicing of CEACAM1 premRNA (23). CEACAM1 has been described as a tumor suppressor being down-regulated in several tumor entities including colon carcinoma, hepatocellular carcinoma, a proportion of breast cancers, bladder cancer, and PCa (24–26). Nonetheless, CEACAM1 appeared overexpressed or neo-expressed in thyroid cancer, gastric cancer, and malignant melanoma (27, 28). One reason for the contradictory expression pattern and functional role of CEACAM1 could be altered CEACAM1-S/CEACAM1-L ratios (22).

In normal adult prostate tissue, CEACAM1 is abundantly expressed on the apical side of glandular prostatic epithelial cells, and its expression is downregulated in PCa (29–31). Additionally, the co-expression of CEACAM20 has been observed and found to be repressed in PCa, indicating its potential involvement in disease progression (32). Furthermore, CEACAM5 has been identified as a validated cell-surface antigen in neuroendocrine PCa, which presents intriguing prospects for its utilization as a therapeutic target (33, 34).

In addition to their potential roles in tumor formation and progression, CEACAMs have been implicated in the modulation of immune response mechanisms as they are expressed on immune cells (22) and serve as pathogen receptors (35–37). Few reports have documented that the stimulation of specific epithelial cells with pro-inflammatory cytokines leads to the up-regulation of several CEACAMs (38, 39), indicating their potential involvement in bridging the gap between inflammation and the establishment of cancer.

To comprehensively investigate the involvement of CEACAMs in prostate inflammation and their potential contribution to the malignant transformation of prostate epithelial cells, we conducted a characterization of CEACAM expression levels in RWPE-1 cells under different culture conditions. Additionally, we correlated these expression levels with the presence of malignancy markers and the migratory properties of the cells. Our study demonstrates that the pro-inflammatory cytokines, tumor necrosis factor alpha (TNFα) and interferon-gamma (IFNγ), induce an up-regulation of CEACAM1 expression in RWPE-1 cells, specifically favoring the CEACAM1-L isoform. This up-regulation is associated with the deregulated expression of JAK/STAT, NFκB, and EMT genes, as well as an increase in cell migration.

## 2 Material and methods

### 2.1 Cell culture

The human prostate epithelial cell line RWPE-1 was purchased from the American Type Culture Collection (ATCC/LGC Standards GmbH, Germany). The cells were maintained in keratinocyte serum-free medium (K-SFM) containing 50 µg/ml bovine pituitary extract and 5 ng/ml epidermal growth factor (Thermo Fisher Scientific, Germany) at 37°C in a humidified 5% CO_2_ atmosphere. For the experiments, serum-starved post-confluent cells were treated with 100 ng/ml TNFα (ImmunoTools, Germany), 100ng/ml IFNγ (ImmunoTools, Germany) and combination for 24 h in K-SFM without supplements. Non-treated cells were used as a control. All experiments were performed with mycoplasma-free cells.

### 2.2 Flow cytometry

RWPE-1 cells (5×10^5^) were stained with 10 μg/ml anti-CEACAM1 (B3-17, A1B domain), anti-CEACAM1 (1/3/5-Sab, N domain), anti-CEACAM5 (5C8C4), anti-CEACAM6 (1H7-4B), anti-CEACAM20 (1-11A), and anti-CEACAM1/3/5/6/8 (6G5j) monoclonal antibodies (mAb) diluted in 3% FCS/PBS for 1 h at 4°C. In the next step the cells were washed with icecold PBS and incubated with FITC conjugated anti-mouse F(ab’)2 (Dianova, Germany) for 30 min at 4°C. Background fluorescence was determined using isotype-matched Ig mAb. The stained cell samples were examined in a FACScalibur flow cytometer (Becton Dickinson, USA) and analyzed by CellQuest Pro^®^ Version 6 (Becton Dickinson, USA). Dead cells identified by propidium iodide staining (1:200 v/v in 3% FCS/PBS) were excluded from the determination.

### 2.3 Real-time PCR

Total RNA extraction was performed with RNAmagic (Bio‐Budget, Germany) in accordance with manufacturer’s instructions. cDNA was synthesized using High‐Capacity cDNA Reverse Transcription Kit (Applied Biosystems, Germany).

RT-PCR was performed using C1000 Touch^®^ Thermal Cycler (BioRad, Germany), 2.5 ng cDNA, 0.5 µM of the respective forward and reverse primers, 0.2 U GoTaq^®^ DNA Polymerase, and 5x GoTaq^®^ reaction buffer (Promega, USA). The thermal conditions were set as followed: 95°C for 5 min followed by 44 cycles at 95°C for 30 sec, 60°C for 45 sec and 72°C for 45 sec and an extension at 72°C for 5 min. PCR products were visualized by agarosis gel electrophoresis using GelRed (1:20 000) and ChemiDoc^®^ Touch Imaging System (Bio-Rad, Germany).

qRT‐PCR was performed using qTOWER³ (Analytik Jena, Germany), specific primers and 5× EvaGreen^®^ QPCR‐Mix II (ROX) (Bio‐Budget, Germany). The thermal cycling conditions were set as followed: 95°C for 15 min followed by 45 cycles of 95°C for 15 sec, 58°C for 30 sec and 72°C for 30 sec. Melting curve analysis was performed for quality control. Evaluation of relative mRNA expression was determined by ΔΔCt method using *GAPDH* and *ACTB* as housekeeping genes. The oligonucleotides sequences are shown in **Supplementary Table S2**.

### 2.4 SDS-PAGE and western blotting

A fraction of the harvested cells was lysed with RIPA buffer (1% Triton X-100, 1% sodium deoxycholate, 0.1% SDS, 150 mM NaCl, 2 mM EDTA, 50 mM sodium fluoride) supplemented with protease inhibitor coctail set III (Merck Millipore, Germany) and PhosSTOP phosphatase inhibitor coctail (Roche, Germany) using Bioruptor^®^ Pico (Diagenode, USA). The protein concentration was determined using the Pierce^®^ BCA Protein Assay Kit (Thermo Fisher Scientific, Germany) according to the manufacturer’s instructions and the protein lysate were compounded with Laemmli buffer (62.5 mM Tris, 2% SDS, 25% glycerol, 0.01% bromophenol blue, 5% β‐mercaptoethanol). Proteins were subjected to Tricine-PAGE, blotted to nitrocellulose membrane (Applichem, Germany) and incubated with 10 µg/ml primary anti-CEACAM1 (1/3/5-Sab, N domain), anti-CEACAM5 (5C8C4), anti-CEACAM6 (1H7-4B) and anti-CEACAM20 (1-11A) mAb followed by HRP-coupled secondary goat anti-mouse Ab (Dianova, Germany). Protein lysate from human CECACAM1 transfected CHO cells (hCC1-CHO) was used as a positive control. RelA and p-RelA was detected using the NF-κB p65 (D14E12) XP^®^ Rabbit mAb, Phospho-NF-kB p65 (Ser536) (93H1) Rabbit mAb and Anti-rabbit IgG HRP-linked Antibody (Cell Signaling, UK) according to the manufacturer’s instructions. Anti-β-actin Ab (Sigma-Aldrich, Germany) was used to confirm equal loading. Proteins were visualized by Clarity^®^ Western ECL Substrate and ChemiDoc^®^ Touch Imaging System (Bio-Rad, Germany).

### 2.5 Immunocytochemistry

RWPE-1 cells were seeded on Karl Hecht Assistant^®^ glass coverslips (Thermo Fisher Scientific, Germany), cultivated until 70-80% confluence and treated with 100 ng/ml TNFα (ImmunoTools, Germany), 100ng/ml IFNγ (ImmunoTools, Germany), and combination of the two cytokines for 24 h in K-SFM without supplements. Cells were fixed with methanol/aceton (1:1 v/v) for 5 min at room temperature (RT) and the background staining was inhibited by incubation with 1% BSA/PBS for one hour at RT. The cells were incubated with 10 µg/ml primary anti-CEACAM1 (C5-1x8), anti-CEACAM5 (5C8C4), anti-CEACAM6 (1H7-4B) and anti-CEACAM20 (1-11A) mAb in 0.5% BSA/PBS overnight at 4°C. Isotype-matched Ig mAb (10 µg/ml) was used as a negative control. A secondary anti-mouse Alexa 488 Ab was applied in combination with 4′,6-Diamidin-2-phenylindol (1:200 v/v, respectively) in 0.5% BSA/PBS and incubated for one hour at RT. After washing with 1x PBS and H2Omilli, the cells were mounted with Fluoromount-G^®^ (SouthernBiotech, USA). For signal detection Nikon Eclipse Ni-E microscope (Nikon, Germany), Ri2 camera (Nikon, Germany) and NIS-Elements version 5.30.02 (Nikon, Germany) were used.

### 2.6 Tissue collection

Postoperative material from patients treated at the Sumy Regional Clinical Hospital between 2020 and 2022 (Departments of Urology; Sumy, Ukraine) was used. This study included six cases of prostatitis and six controls obtained from patients after transrectal prostate needle biopsy or transurethral resection of the prostate. Prostatitis was confirmed by at least two pathologists. The patients received written study information from their treating physician and provided written informed consent for tissue investigation. The written informed consent was kept in the patient’s file (Inpatient Health Record). The Institutional Review Board of the Academic and Research Medical Institute of Sumy State University (Sumy, Ukraine) approved the study design (№ 05/3-2022), which adhered to ethical guidelines for experimental and clinical research.

### 2.7 Immunohistochemistry

#### 2.7.1 DAB staining

Serial sections of 4 µm were prepared from paraffin-embedded tissue previously fixed in neutrally buffered formalin and mounted on 3-aminopropyltriethoxysilane-coated slides. The tissue slices were deparaffinized with xylene and rehydrated in a descending alcohol series (100%, 96% and 70%). Heat-mediated antigen retrieval was performed in 0.01 M sodium citrate buffer for 30 min at 97°C. Endogenous peroxidase activity was blocked by treating the samples with 3% H2O2 for 5 min. Background staining was inhibited by incubation with 1% BSA/PBS for one hour at RT. The tissue slices were incubated with 10 µg/ml anti-CEACAM1 (C5-1x8), anti-CEACAM5 (5C8C4), anti-CEACAM6 (1H7-4B) and anti-CEACAM20 (1-11A) mAbs in 0.5% BSA/PBS overnight at 4°C. Isotype-matched Ig mAb (10 µg/ml) was used as a negative control. A biotinylated secondary rabbit anti-mouse Ab (Dako, Germany) was applied 1:200 v/v in 0.5% BSA/PBS and incubated for one hour at RT. After washing, the tissue slices were incubated with VECTASTAIN ABC reagent (Vector Laboratories, USA) for 30 min according to the manufacturer’s instructions. The staining was visualized using diaminobenzidine (DAB) substrate, and the color intensity was monitored using light microscopy. The DAB reaction was stopped with distilled H2O as soon as the desired color intensity was achieved. DAB-negative structures were identified by additional counterstaining with hematoxylin. Finally, slices were dehydrated in ascending alcohol series/xylene and mounted with Xylene Substitute Mountant (Thermo Fisher Scientific, Germany). For signal detection Nikon Eclipse Ni-E microscope (Nikon, Germany), Ri2 camera (Nikon, Germany) and NIS-Elements version 5.30.02 (Nikon, Germany) were used.

#### 2.7.2 Immunofluorescence staining

The 3 µm sections of paraffin-embedded tissue were deparaffinized and rehydrated using graded alcohol and xylene. To prevent autofluorescence from endogenous fluorophores, MaxBlock^®^ Autofluorescence Reducing Reagent Kit (Dianova, Germany) was employed, followed by washing with 60% ethanol and distilled water. Heat-mediated antigen retrieval, background staining prevention, incubation with primary antibodies, and biotinylated secondary antibody incubation were performed as previously described. The double staining procedure was carried out sequentially, with the first staining using rabbit polyclonal anti-CEACAM1 antibody. Subsequently, Cy3-labeled Streptavidin was applied, followed by overnight incubation at 4°C with mouse monoclonal anti-CD45 antibody. Mouse and rabbit IgG control antibodies were used as negative controls, respectively. For visualization of receptor-positive green signals and general tissue structure, Alexa 488 with DAPI, diluted in 0.5% BSA/PBS (1:200), was applied. Before final mounting using Fluoromount-G^®^ (SouthernBiotech, USA), a MaxBlock^®^ Post-Detection Conditioner Kit (Dianova, Germany) was utilized. It was crucial to employ primary antibodies from different species (mouse and rabbit) to avoid cross-reactivity between them.

### 2.8 Gap closure migration assay

RWPE-1 cells (56.000 cells/well) were seeded in Ibidi chambers (Culture-Inserts 2 well for self-insertion, Cat. No. 80209) on a 24 well plate (Greiner Bio-One, Germany) and incubated overnight. The following day, inserts were removed to create a gap and cell patches were washed with 1x PBS, before adding 2 ml of the respective medium (without cytokines, +TNFα, +IFNγ, +TNFα+IFNγ) per well. Three fields of view per well in three replicate wells were monitored. Time-lapse imaging with ImageXpress^®^ Pico (Molecular Devices, USA) and Cell Reporter Xpress 2.9 software was set up for acquisition every hour for 24 h in total. Images were analyzed using ImageJ 1.53t software. In total three fields of view per well from three replicate well for each experiment were analyzed.

### 2.9 Transfection

The pcDNA3.1 neo (-) plasmid encoding human CEACAM1-4L was transfected into RWPE-1 cells using FuGene^®^ according to the manufacturer’s instructions (Promega). RNA isolation and gap closure assay were performed on day 3 posttransfection.

### 2.10 Statistics

All data are shown as mean ± SEM with n = 3. Statistical analysis was performed using GraphPad Prism (Vers.9, Statcon GmbH, Germany). One-way ANOVA followed by Bonferroni *post hoc* analysis was used. Expression data of qRT-PCR were analyzed using anti-logarithmic data. Values of *p< 0.05, ** p< 0.01, *** p< 0.001 and # p< 0.0001 were considered statistically significant.

## 3 Results

### 3.1 TNFα and IFNγ increase CEACAM1 expression in RWPE-1 cells

In order to mimic an inflammation of prostate epithelial cells, RWPE-1 cells were subjected to various treatments, including lipopolysaccharide (LPS), interleukin-6 (IL-6), interleukin-8 (IL-8), interleukin-17 (IL-17), TNFα, and IFNγ. The individual and combined effects of these cytokines were evaluated (**Supplementary Figure 1**). Among all the treatments, only TNFα and IFNγ showed significant alterations in the expression of CEACAMs in RWPE-1 cells. Consequently, a treatment with TNFα and IFNγ at a concentration of 100 ng/ml for a duration of 24 h was selected for further experiments due to their predisposition to induce changes in CEACAM expression.

**Figure 1:**
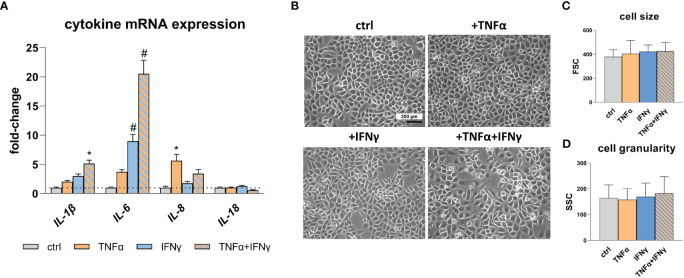
Pro-inflammatory cytokine expression and RWPE-1 cell morphology. **(A)** Increased *IL-1β*, *IL-6* and *IL-8* mRNA expression after TNFα, IFNγ and TNFα+IFNγ treatment (100ng/mL, 24h) relative to the untreated control (ctrl; dotted line: fold-change 1) detected by qRT-PCR. *GAPDH* (Glycerinaldehyd-3-phosphat-Dehydrogenase) and *ACTB* (β-actin) were used as reference genes, efficiency corrected Ct method. **(B)** Representative microscopic images show unaltered cell morphology after TNFα, IFNγ and combination treatment; scale bar = 200 µm, magnification is the same in all four photographs. **(C)** Cell size and **(D)** cell granularity measured by flow cytometry remained unaffected upon TNFα, IFNγ and combined treatment. Data are represented as mean ± SEM, n=3, one-way ANOVA followed by Bonferroni *post hoc* analysis, *p < 0.05, ^#^ *p* < 0.0001.

To assess the inflammatory state of the cells following cytokine treatment, gene expression analysis of endogenous pro-inflammatory cytokines was performed using quantitative real-time polymerase chain reaction (qRT-PCR). Specifically, the expression levels of *IL-1β*, *IL-6*, *IL-8*, and *IL-18* were analyzed (**Figure 1A**). Treatment with TNFα results in an increased expression of *IL-8*, while IFNγ treatment leads to elevated expression of *IL-1β* and *IL-6*. Notably, the most significant effect is observed for *IL-1β* (*p*<0.05) and *IL-6* (*p*<0.0001) when TNFα and IFNγ is used in combination, indicating a synergistic effect. No change in *IL-18* expression is detected after stimulation with TNFα and IFNγ. Cell morphology (**Figure 1B**), cell size (**Figure 1C**), and cell granularity (**Figure 1D**) remains unaffected by the treatment with TNFα and IFNγ.

**Figure 2:**
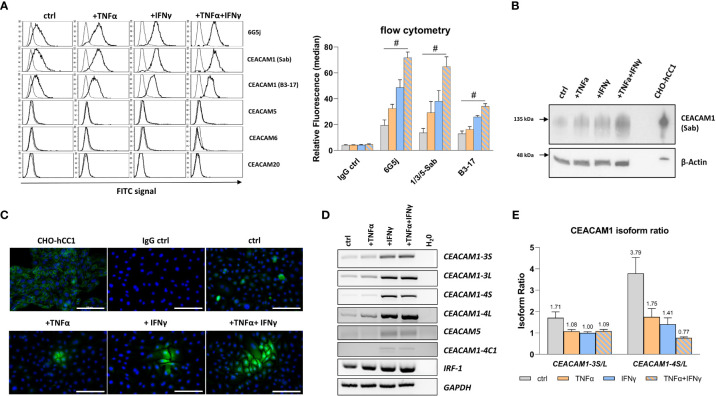
TNFα and IFNγ increase CEACAM1 expression in RWPE-1 cells. **(A)** Flow cytometry analysis shows increased fluorescence signal for CEACAM1 (6G5j, Sab, and B3-17 mAbs) on the surface of RWPE-1 cells. mAb stainings are indicated with a black line, negative control staining (mouse IgG) is indicated with a grey line. **(B)** Western blot analysis shows increased CEACAM1 (120 kDa, Sab mAb) protein expression after TNFα, IFNγ and combination treatment compared to the control. Positive control: protein lysate isolated from human-CEACAM1 transfected CHO cell line (CHO-hCC1), loading control: β-actin (42 kDa). **(C)** Immunofluorescence staining of untreated RWPE-1 cells shows single spread CEACAM1 positive cells (C5-1X mAb). After TNFα and IFNγ treatment small groups of cells are positive for CEACAM1, which is increased after the combined treatment with TNFα and IFNγ. Positive control: CHO-hCC1; negative control: mouse IgG; scale bar=100 µm. **(D)** Increased mRNA expression of *CEACAM1-3S, CEACAM1-3L, CEACAM1-4S, CEACAM1-4L* and *IRF-1* in RWPE-1 cells after TNFα treatment compared to untreated cells. IFNγ and combined treatment with TNFα and IFNγ increases additionally *CEACAM5* and *CEACAM1-4C1* mRNA expression. Representative agarosis gel electrophoresis of RT-PCR products using *GAPDH* as a reference gene and H_2_O as a no template control. **(E)** The *CEACAM1-3S/L* and *CEACAM1-4S/L* isoform ratio is decreased after TNFα, IFNγ and combined treatment compared to the control. CEACAM1 isoform ratio was calculated from band intensity using ImageJ software. Data are represented as mean ± SEM, n=3, one-way ANOVA followed by Bonferroni *post hoc* analysis, ^#^ *p* < 0.0001.

CEACAM protein expression in RWPE-1 cells was analyzed using flow cytometry, immunocytochemistry, and western blot. The initial flow cytometry analysis using the polyspecific 6G5j mAb reveals an expression of CEACAMs in non-treated cells. Upon treatment of RWPE-1 cells with TNFα and IFNγ a synergistic increase of CEACAM expression is observed (**Figure 2A**). In order to identify the specific CEACAMs contributing to the signal, further mAbs were used. Notably, an equal signal is detected with Sab mAb, which detects the N-domain of CEACAM1, suggesting that the signal detected by 6G5j mAb is likely attributed exclusively to CEACAM1 (**Figure 2A**). Interestingly, when B3-17 mAb, which recognizes the A1B1-domains of CEACAM1, was used in flow cytometry, a lower signal compared to the Sab mAb was observed (**Figure 2A**). Furthermore, immunocytochemistry (**Figure 2B**) and western blot analysis (**Figure 2C**) demonstrates elevated CEACAM1 expression in response to TNFα and IFNγ treatment in an additive manner. In contrast, CEACAM5, CEACAM6, and CEACAM20 are not detected in RWPE-1 cells (data not shown).

### 3.2 Decreased CEACAM1-S/CEACAM1-L ratio upon inflammation stimulus

CEACAM1 has a number of membrane anchored or soluble isoforms. The membrane anchored CEACAM1 splice variants include 1–4 ectodomains with either short (S) or long (L) cytoplasmic domain. As the optimal CEACAM1-S/CEACAM1-L ratio is important for maintaining normal cellular function, the gene expression of the most abundant membrane anchored CEACAM1 splice variants CEACAM1-3S, CEACAM1-3L, CEACAM1-4S, and CEACAM1-4L was analyzed. The mRNA expression was investigated using reverse transcription-polymerase chain reaction (RT-PCR), while the gene products were separated and visualized using agarose gel electrophoresis (**Figure 2D**). TNFα and IFNγ treatment induces the mRNA expression of *CEACAM1-3S*, C*EACAM1-3L*, and *CEACAM1-4L*. Additionally, IFNγ leads to a neo-expression of the soluble CEACAM1 isoform *CEACAM1-4C1* as well as *CEACAM5*. Interferon regulatory factor-1 (*IRF-1*), a transcriptional activator of CEACAM1, is also up-regulated by the cytokine treatment. The CEACAM1-S/CEACAM1-L ratio is decreased by TNFα (CEACAM1-3: 1.71 to 1.08; CEACAM1-4: 3.79 to 1.75) and IFNγ (CEACAM1-3: 1.71 to 1.0; CEACAM1-4: 3.79 to 1.41) accompanied by a shift towards the CEACAM1-L isoform. Notably, TNFα+IFNγ causes even a reversal of the CEACAM1-S/CEACAM1-L ratio specifically for the CEACAM1-4 variant (3.79 to 0.77) (**Figure 2E**). Soluble CEACAM1 isoforms are not detected in cell culture supernatants (data not shown).

### 3.3 Intracellular localization of CEACAM1 in prostatitis tissue

**Figure 3:**
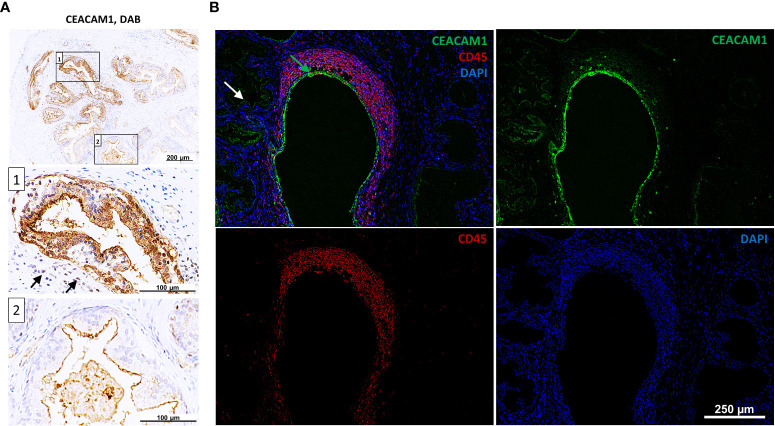
Intracellular staining of CEACAM1 in inflamed human prostatic tissue. **(A)** Immunohistochemical DAB (3,3’-Diaminobenzidine) staining indicates intracellular CEACAM1 expression (box 1) near the immune infiltrate (indicated with arrows). Distant from inflamed tissue areas the CEACAM1 expression is associated with the apical side of prostatic gland epithelium (box 2); scale bar=100/200 μm. These results were confirmed in immunofluorescence staining **(B)**. Within the inflamed areas (leukocyte staining with anti-CD45 mAb, red) the CEACAM1 staining (green) is located intracellularly and surrounds the nuclei (DAPI, blue). Intracellular staining: green arrow, apical staining: white arrow; scale bar=250 μm. Representative microscopic images from n=6.

The expression of CEACAMs was examined in prostate tissue samples (n=12) collected from patients who underwent transrectal prostate needle biopsy or transurethral resection of the prostate. The prostatitis tissue sections (n=6) contained regions with leukocytes infiltrates as well as non-infiltrated tissue areas, which were used as an internal control. Normal prostate tissue (n=6) was used as an external control (**Supplementary Figure 2**). Alterations in the subcellular localization of CEACAM1 depending on the side of inflammation are notable. Specifically, a redistribution of CEACAM1 from the plasma membrane to the cytoplasm in the areas infiltrated by leukocytes (**Figures 3A, B**; box 1; leukocytes indicated with black arrows) is observed, whereas apical CEACAM1 expression is observed in non-infiltrated areas (**Figures 3A, B**; box 2). Similar findings are obtained using DAB (3,3’-Diaminobenzidine) and Immunofluorescence (IF). CEACAM20 is also detected on the apical side of prostate epithelial cells. However, while CEACAM1 exhibits a stronger signal and is continuously present in every gland, CEACAM20 displays a relatively weak signal and is not present in every gland (**Supplementary Figure 3**). CEACAM5 and CEACAM6 are not detectable in neither DAB nor IF staining (data not shown).

### 3.4 Increased cell migration and expression of malignancy markers are associated with CEACAM1-L overexpression during inflammation

**Figure 4:**
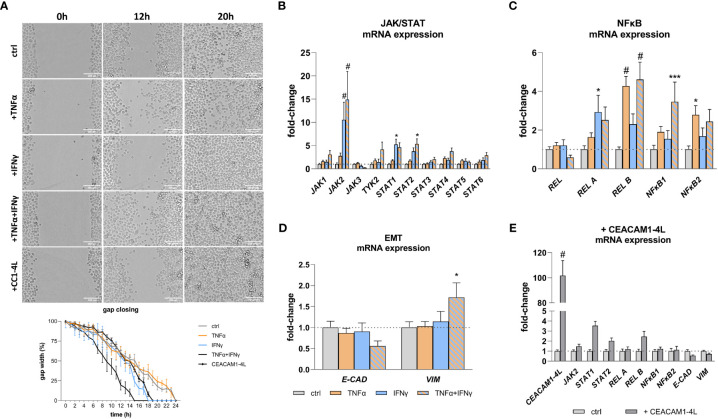
Increased cell migration and malignancy marker expression after TNFα and IFNγ treatment. **(A)** Gap closure assay shows increased migration of RWPE-1 cells after IFNγ and TNFα+IFNγ treatment, as well as CEACAM1-4L transfection. Scale bar=200 μm. qRT-PCR analyses of malignancy markers show significantly increased mRNA expression of **(B)** JAK/STAT pathway genes *JAK2* and *STAT1* after IFNγ treatment. TNFα+IFNγ treatment increased *JAK2* and *STAT2* expression. **(C)** NFκB pathway genes *REL B* and *NFκB2* were up-regulated by TNFα. TNFα+IFNγ treatment increased *REL B* and *NFκB1* expression. **(D)** The mRNA expression of the epithelial-mesenchymal transition markers *E-CAD* and *VIM* is deregulated compared to the untreated controls after TNFα+IFNγ treatment. **(E)** Overexpression of CEACAM1-4L in RWPE-1 cells leads to deregulated *JAK2, STAT1, STAT2, REL B*, *E-CAD* and *VIM* expression. *GAPDH* (Glycerinaldehyd-3-phosphat-Dehydrogenase) and *ACTB* (β-actin) were used as reference genes, efficiency corrected Ct method. Dotted line represents untreated cells (ctrl), fold-change 1. Data are represented as mean ± SEM, n=3, one-way ANOVA followed by Bonferroni *post hoc* analysis, *p < 0.05, ***p < 0.001, ^#^ *p* < 0.0001.

Pro-inflammatory cytokines and CEACAM1 have been implicated in the modulation of cellular functions and signaling pathways, contributing to the expression of critical mediators in cancer and inflammation. The findings of this study demonstrate that TNFα and IFNγ treatment results in a deregulated expression of JAK/STAT (**Figure 4B**) and NFκB (**Figure 4C**) pathway genes, as well of genes involved in EMT (**Figure 4D**). Moreover, the protein expression as well as phosphorylation of the NFκB pathway member RelA are increased when treated with TNFα and IFNγ in a synergistic manner (**Supplementary Figure 4**). Additionally, gap closure assay reveals an increased migration after IFNγ treatment (gap closure: 18h vs. 24h), which is further enhanced by TNFα (gap closure: 15h vs 24) (**Figure 4A**). To test the hypothesis that overexpressed CEACAM1-L isoform upon an inflammation stimulus may lead to an increased cell migration and deregulation of the expression of the malignancy markers in RWPE-1 cells we transfected the cells with human CEACAM1-4L and analyzed the parameters, respectively. The transfection leads to a deregulation of *STAT1, STAT2, REL B, E-CADH*, and *VIM* in CEACAM1-4L transfected cells compared to the controls (**Figure 4E**). Furthermore, the cell migration is enhanced in CEACAM1-4L transfected cells compared to the controls (gap closure: 19h vs 24h). The effect is comparable to the IFNγ treatment (**Figure 4A**).

## 4 Discussion

Several studies reported deregulated CEACAMs in PCa (29–34) and the there is an evidence of deregulated CEACAMs in inflammation (38, 39), indicating their potential involvement in bridging the gap between inflammation and the tumorigenesis. In this study, we investigated the expression of CEACAMs in an *in-vitro* prostatitis model and the potential role of CEACAMs in malignant transformation of prostate epithelial cells.

In accordance with previous findings, we have successfully validated the expression of CEACAM1 in RWPE-1 cells and prostate tissue. Interestingly, the expression of CEACAM20 was detected in prostate tissue, but not in RWPE-1 cells. In contrast to the observations reported by Zhang et al. (32), we did not detect a concurrent co-expression of CEACAM1 and CEACAM20 in prostate tissue. While CEACAM1 and CEACAM20 are restricted to the apical membrane of glandular prostate epithelial cells, the expression of CEACAM20 is not observed in every gland as it is the case for CEACAM1. This discrepancy may account for the absence of CEACAM20 in RWPE-1 cells, as the cells used in this study might lack the expression of CEACAM20. Interestingly, immune infiltrated areas of prostate tissue revealed also an intracellular staining of CEACAM1 indicating enhanced CEACAM1 synthesis and storage within the cells. Intracellular CEACAM1 may possess additional functions beyond its involvement in cell-cell communication and signaling, potentially exerting effects on the cell itself through intracellular signaling.

Our findings demonstrate an up-regulation of CEACAM1 in response to a treatment with TNFα and IFNγ in RWPE-1 cells associated with induced *IRF-1* expression. However, we observed no up-regulation of CEACAMs upon treatment with LPS, IL-6, IL-8, and IL-17 (data not shown). TNFα and IFNγ are pleiotropic Th1-type cytokines that play crucial roles in modulating immune and inflammatory responses, as well as contributing to pathogenesis at aberrant expression levels (40, 41). Both cytokines induce IRF-1 (40, 42), which binds to Interferon-Stimulated Response Element (ISRE) in IFNγ-inducible gene promotors thereby activating the expression of genes involved in immune response, cell growth, apoptosis, tumor suppression, or tumorigenesis (43, 44). Previous studies have shown that CEACAM1 possesses an ISRE in its promoter region and is synergistically induced by TNFα and IFNγ in endothelial cells, colorectal carcinoma cells, cervix carcinoma cells, and breast carcinoma cells (45–48). In this study, we provide confirmation of these findings for the first time using non-malignant epithelial cells.

Twelve CEACAM1 splice variants with different features can be expressed in human cells, including membrane anchored or soluble isoforms. The membrane anchored CEACAM1 splice variants include 1-3 or 1-4 extracellular Ig-like domains with either short (S) or long (L) cytoplasmic domains (49). Different CEACAM1-isoforms are most frequently co-expressed by the same cell and their ratio determine the outcome of cellular signaling (50, 51). While RWPE-1 cells predominantly express the CEACAM1-S isoforms as already reported for non-malignant prostate tissue by Gaur et al. (23), the treatment with TNFα and IFNγ increases the expression of splice variants *CEACAM1-3S*, *CEACAM1-3L*, *CEACAM1-4S*, and *CEACAM1-4L* with a shift to the CEACAM1-L isoform. The most prominent effect was observed for CEACAM1-4 with an even reversed CEACAM1-S/CEACAM1-L ratio. Similar results of alternative splicing towards the CEACAM1-L isoform upon IFNγ treatment were reported by Dery et al. for breast cancer cells (40). It is assumed that during inflammation the induction of IRF-1 leads to the expression of CEACAM1-L isoform, which if chronically expressed may promote malignant transformation of epithelial cells. Indeed, overexpression of CEACAM1-L variant is linked to melanoma progression and metastasis (52) and tumorigenesis in breast tissue (23). Due to the high heterogeneity of PCa, the CEACAM1-S/CEACAM1-L ratio varies among different subsets of cancer cells (23). However, a transfection of PCa cell line DU145 with CEACAM1-L decreased the tumorigenic potential in a xenograft animal model, suggesting that CEACAM1 is a tumor suppressor in PCa (24). Therefore, the widely accepted tumor suppressor activity of CEACAM1-L in PCa should be reevaluated by analyzing different PCa cell lines, considering factors such as androgen sensitivity, neuroendocrine characteristics, and the given CEACAM1-S/CEACAM1-L ratio. Nevertheless, increased expression of the CEACAM1-L isoform in normal prostate epithelial cells upon inflammation stimulus as shown in this study may lead to an early onset of malignant transformation.

To investigate the potential role of CEACAM1-L in inflammation as a potential initiator of malignant transformation, transfected RWPE-1 cells were transfected with human CEACAM1-4L and the expression of malignancy markers compared to the non-transfected, cytokine-treated cells were analyzed. The overexpression of CEACAM1-L in glioblastoma-initiating cells has been associated with increased tumorigenesis through the activation of the STAT3 signaling pathway (53). Additionally, it has been reported that CEACAM1 relates to the activation of the non-canonical NFκB pathway (54) and controls the EMT switch in murine mammary carcinoma (55). Thus, we analyzed the gene expression of JAK/STAT pathway genes, NFκB pathway genes, as well as genes involved in EMT. On the one hand, the treatment of RWPE-1 cells with TNFα and IFNγ results in an increase of the expression of not only CEACAM1 but also of *JAK2, STAT1, STAT2, REL A, REL B, NFκB1, NFκB2*, and *VIM*. Additionally, a decrease in the expression of *E-CAD* is observed. The deregulated expression of malignancy markers after cytokine treatment could be verified at the protein level by examining exemplary REL A and its phosphorylated form. On the other hand, RWPE-1 cells transfected with human CEACAM1-4L exhibit an increased expression of *STAT1, STAT2, REL B* and a decreased expression of *E-CAD* and *VIM*, indicating that the deregulation of genes associated with malignancy can be attributed to the overexpression of CEACAM1-4L. However, one functional principle of CEACAM1 is that it barely does anything on its own but influences many other molecules in their function (22) and represent only a part of a complex mechanism contributing to carcinogenesis. As malignant transformation of the cells is associated with increased migration, the migration properties of RWPE-1 cells after cytokine treatment versus CEACAM1-4L transfected cells were analyzed in this study. Increased migration is observed after IFNγ treatment, which is enhanced by TNFα. CEACAM1-4L transfected RWPE-1 cells show also increased migration compared to the untreated controls. These findings support and further emphasize the significance of CEACAM1-4L in the context of inflammation-related oncogenesis.

Furthermore, we could observe a neo-expression of the soluble CEACAM1 isoform *CEACAM1-4C1*, and *CEACAM5* upon IFNγ treatment of RWPE-1 cells. Due to homophilic and heterophilic dimerization of CEACAM1, the soluble CEACAM1 can serve as a ligand for CEACAM1, CEACAM5, CEACAM6 or CEACAM8 on epithelial cells, immune cells, and endothelia promoting different functions, e. g. angiogenesis, activation/inactivation of immune cells, proliferation and migration (56–63). CEACAM5 leads to angiogenesis, tumor formation, metastasis, modulation of immune cells, and is a wide accepted tumor marker for different tumor entities including neuroendocrine PCa (33, 64). Thus, elevated levels of soluble CEACAM1 as well as membrane-anchored or soluble CEACAM5 proteins can modify immune response, angiogenesis and properties of epithelial cells favoring tumor-appropriated environment. However, no corresponding protein expression was observed in the RWPE-1 cells. Thus, soluble CEACAM1 and CEACAM5 most likely do not participate in PCa initiation and progression.

## 5 Conclusion

This study provides groundbreaking insights into the involvement of CEACAMs, specifically CEACAM1 and its isoforms, in the inflammatory response of prostate epithelial cells and their potential contribution to malignant transformation. We demonstrate that TNFα and IFNγ stimulation leads to an up-regulation of CEACAM1 expression, predominantly favoring the CEACAM1-L isoform. This isoform shift correlates with deregulated expression of genes associated with the JAK/STAT and NFκB signaling pathways, as well as genes involved in EMT, and enhanced cell migration. Our findings provide supporting evidence for the hypothesis that CEACAM1-4L may synergistically contribute to inflammation-induced oncogenesis in the prostate.

## Data availability statement

The raw data supporting the conclusions of this article will be made available by the authors, without undue reservation.

## Ethics statement

The studies involving humans were approved by Institutional Review Board of the Academic and Research Medical Institute of Sumy State University (Sumy, Ukraine). The studies were conducted in accordance with the local legislation and institutional requirements. The participants provided their written informed consent to participate in this study.

## Author contributions

Conception, IK. Methodology, IK. Formal analysis, IK, GW. Investigation, IK, ML. Resources, IK, MW, GW. Data curation, IK. Writing - original draft preparation, IK. Writing - review and editing, IK, ML, MW and GW. Project administration, IK, GW. Acquisition, GW. All authors contributed to the article and approved the submitted version.

## In Memoriam

This work is dedicated to Bernhard B. Singer. With his passing, we have lost an extraordinary friend, a passionate mentor, and an enthusiastic scientist. Bernhard B. Singer’s impact on the CEACAM field was unparalleled. It was a privilege to have had the opportunity to know and learn from him. We are determined to continue his legacy by building upon his lifetime achievements to the best of our abilities.
