# Supplementary material for: CEACAM expression in an in-vitro prostatitis model
Source: Front Immunol. 2023 Aug 25;14:1236343. doi: 10.3389/fimmu.2023.1236343 (PMC10485834; doi:10.3389/fimmu.2023.1236343)
Supplement: Supplementary file 1 [file DataSheet_1.docx]

Supplementary Material

CEACAM expression in an *in-vitro* prostatitis model

**Irina Kube-Golovin^1^, Mykola Lyndin^1,2^, Marc Wiesehöfer^1^, Gunther Wennemuth^1*^**

^1^ Department of Anatomy, University Clinic Essen, Medical Faculty, Hufelandstrasse 55, D-45147 Essen

^2^ Academic and Research Medical Institute, Department of Pathology, Sumy State University, Sumy, 40000, Ukraine

*** Correspondence:**Gunther Wennemuth
[Gunther.Wennemuth@uk-essen.de](mailto:Gunther.Wennemuth@uk-essen.de)

# Supplementary Data

## Antibodies

**Suppl. Table S1:** Primary antibodies used for flow cytometry, WB and IF analyses.

| **Ab** | **Protein** | **Host** | **Specificity** | **Supplier/ Cat. No.** |
| --- | --- | --- | --- | --- |
| Anti-β-actin | β-actin | mouse | human | Sigma-Aldrich, Hamburg, Germany  A5441-100UL |
| CD-45 | CD-45 | mouse | human | Dako, Glostrup, Denmark  M0701 |
| B3-17 | CEACAM1 | mouse | human | Singer, Essen, Germany |
| C5-1X | CEACAM1 | mouse | human | Singer, Essen, Germany |
| CC1/3/5-Sab | CEACAM5 | mouse | human | LeukoCom, Essen, Germany |
| 5C8C4 | CEACAM6 | mouse | human | LeukoCom, Essen, Germany |
| 1H7-4B | CEACAM20 | mouse | human | LeukoCom, Essen, Germany |
| 1-11A | CEACAM1/3/5 | mouse | human | LeukoCom, Essen, Germany |
| 6G5j | CEACAM1/3/5/6/8 | mouse | human | LeukoCom, Essen, Germany |
| NF-κB p65 (D14E12) XP® Rabbit mAb | RelA | rabbit | human | Cell Signaling, Cambridge, UK  #8242 |
| Phospho-NF-κB p65  (Ser536) (93H1) | p-RelA | rabbit | human | Cell Signaling, Cambridge, UK  #3033 |

## Primers

**Suppl. Table S2:** Oligonucleotides used for amplification of genes by quantitative RT-PCR. Oligonucleotides were designed using PrimerBlast (NCBI) and synthesized by Eurofins (Eurofins MWG Synthesis, Ebersberg, Germany).

| **Gene** | **Sequence (5`🡪 3`) For** | **Sequence (3`🡪 5`) Rev** |
| --- | --- | --- |
| ***ACTB*** | GCTCGTCGTCGACAACGGCTC | CAAACATGATCTGGGTCATCTTCTC |
| ***CEACAM1-3S*** | CAAGACGATCATAGTCACTGATAA | GGAGTGGTCCTGAGCTGC |
| ***CEACAM1-3L*** | CAAGACGATCATAGTCACTGATAA | TGGAGTGGTCCTGAGTGTG |
| ***CEACAM1-4S*** | AGACGATCATAGTCACTGAGCT | GGAGTGGTCCTGAGCTGC |
| ***CEACAM1-4L*** | AGACGATCATAGTCACTGAGCT | TGGAGTGGTCCTGAGTGTG |
| ***CEACAM1-4C1*** | AAGACGATCATAGTCACTGAGCT | TTGCACACCATTGACAGAGT |
| ***CEACAM5*** | GCCTCAATAGGACCACAGTCAC | CAGGTTAAGGCTACAGCATCCTC |
| ***E-CAD*** | GAGAACGCATTGCCACATACAC | GGGTCTGTCATGGAAGGTGC |
| ***GAPDH*** | TCAAGGCTGAGAACGGGAAG | TGGACTCCACGACGTACTCA |
| ***IL-1β*** | AAATGATGGCTTATTACAGTGGCA | GTGGTGGTCGGAGATTCGTA |
| ***IL-6*** | CAGCCCTGAGAAAGGAGACAT | GGTTCAGGTTGTTTTCTGCCA |
| ***IL-8*** | TGGAGAAGTTTTTGAAGAGGGCT | CAACAGACCCACACAATACATGAAG |
| ***IL-18*** | ACCTTCCAGATCGCTTCCTC | CCAGGTTTTCATCATCTTCAGCTAT |
| ***IRF-1*** | ACCCTGGCTAGAGATGCAGATT | GCTTTGTATCGGCCTGTGTGA |
| ***JAK1*** | TGCTTTGAGAAGTCTGAGCAGG | CTGCTTCTTGAGGTGGCTCAT |
| ***JAK2*** | TGGTCGCCCGATCTGTGTA | TTCTGTCATCGTAAGGCAGGC |
| ***JAK3*** | CTCGTGATGGGCTGTGGAAT | CTGCCAAAGTTGCCCTTGC |
| ***NFkB1*** | TTCGGATAGTTTCGGCGGTG | GGGAAGCTATACCCTGGACCT |
| ***NFkB2*** | CACGTACCGACAGACAACCT | TCTTCCTTCACCTCTGCTGTG |
| ***REL*** | ACCCAATTTATGACAACCGTGC | ATGCCTTTTGCTTCCCAATCG |
| ***REL A*** | GCTGCATCCACAGTTTCCAGA | TCCCCACGCTGCTCTTCTAT |
| ***REL B*** | ACAAGGTGCAGAAAGAGGACAT | TGTCACGGGCTCGACAATC |
| ***STAT1*** | GTTATGGGACCGCACCTTCA | AGTGAACTGGACCCCTGTCT |
| ***STAT2*** | CTGAACTATGAGTGTGGCCGT | GATCCTGGGAAAAGGGCTGAAT |
| ***STAT3*** | TTATCAGTAAGGAGCGGGAGC | TCTGGGTCTTACCGCTGATG |
| ***STAT4*** | CTCAGCCTTGCGAAGTTTCAA | ACACCGCATACACACTTGGA |
| ***STAT5*** | GAAGCAGGTGTCTCTGGAGG | TCTCGGCCAACTTCTCACAC |
| ***STAT6*** | AGCCCAAGGATGAGGCTTTC | AGTGGTTGGTCCCTTTCCAC |
| ***TYK2*** | TCAAAGCTGCATCCCTTCTGG | CCTCTGGTAGAAATGCTCCTTCTTC |
| ***VIM*** | CTCCCTGAACCTGAGGGAAAC | GCAGTTTTTCAGGAGCGCAAG |

# Supplementary Figures


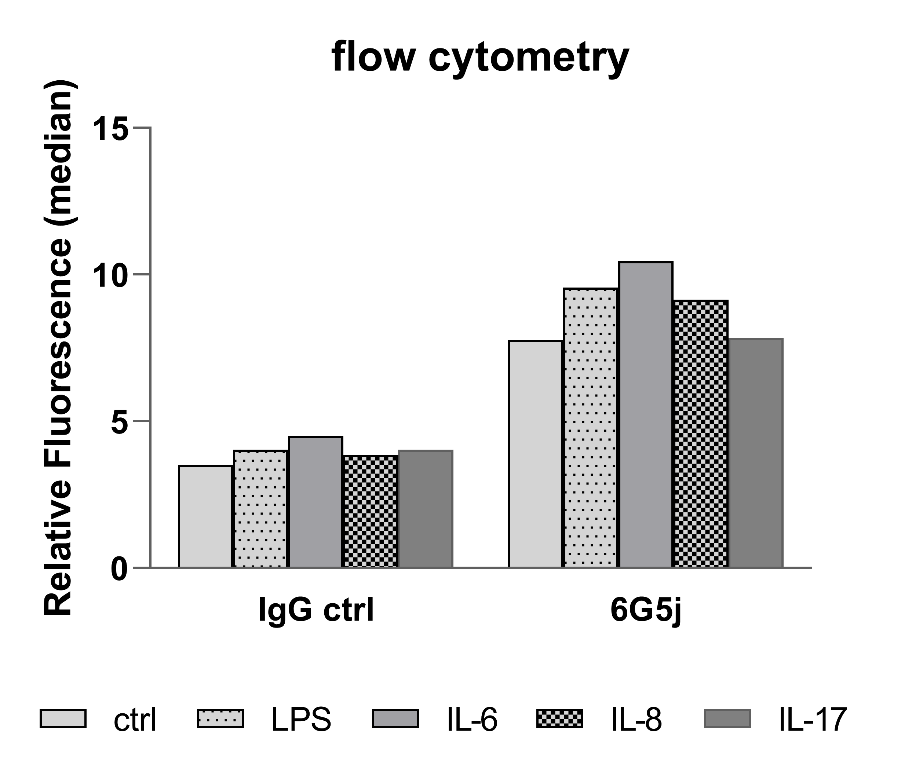


**Supplementary Figure 1.** **CEACAM expression after LPS, IL-6, IL-8, and IL-17 treatment.** Flow cytometry analysis shows no differences in fluorescence signal for CEACAMs on the RWPE-1 cells after treatment with LPS, IL-6, IL-8, and IL-17 (100ng/mL, 24h).


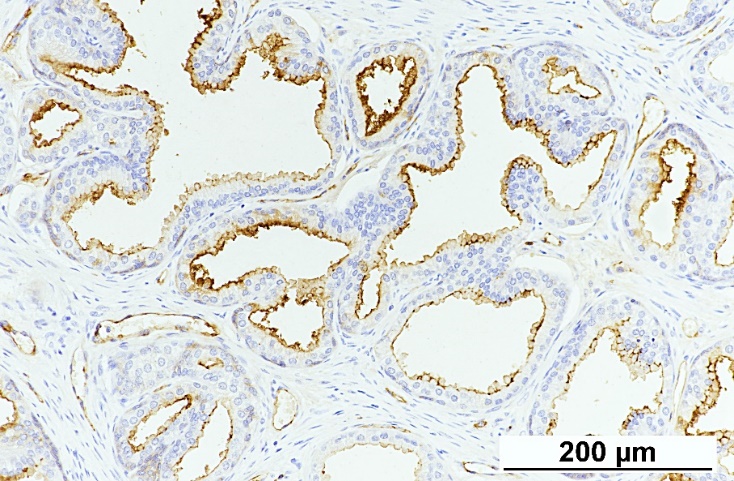

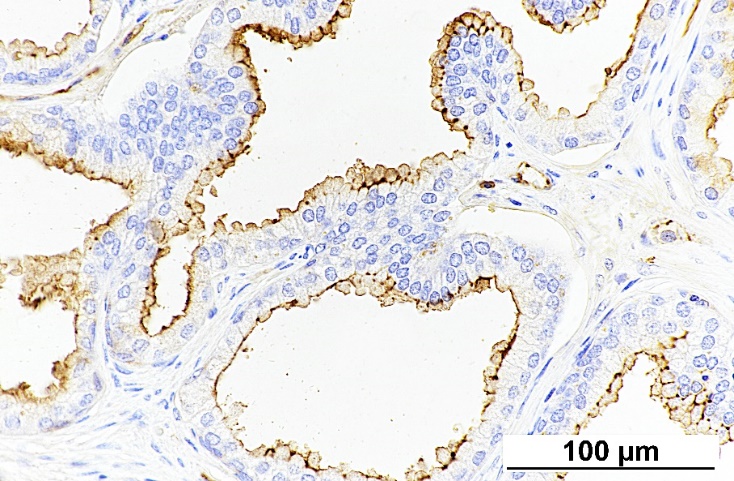


**CEACAM1**

**CEACAM1**

**Supplementary Figure 2.** **CEACAM1 staining in human prostate tissue.** Immunohistochemical DAB staining shows apical CEACAM1 expression. Representative microscopic images from n=6, scale bar: 100/200 μm.

**
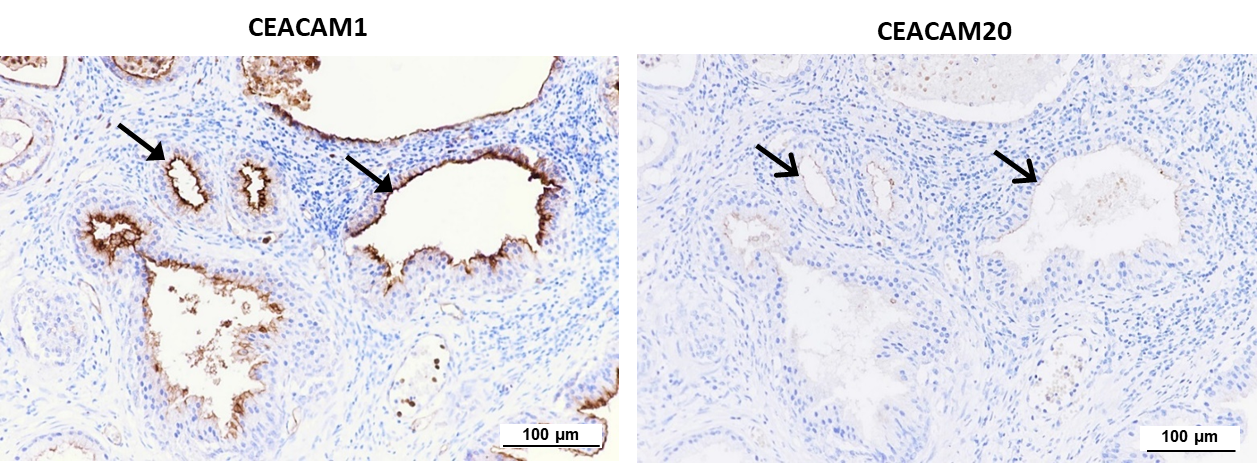
**

**Supplementary Figure 3.** **CEACAM1 and CEACAM20 staining in human prostate tissue.** Immunohistochemical DAB staining shows CEACAM1 (A) and CAECAM20 (B) expression (indicated with the black arrows). Representative microscopic images from n=6, scale bar: 100 μm.


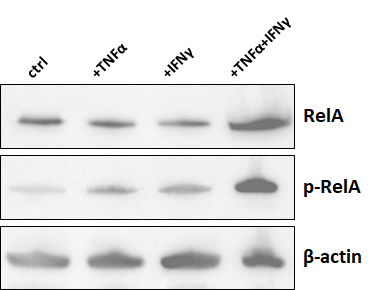


**Supplementary Figure 4.** **RelA protein expression and phosphorylation after cytokine treatment**. Western Blot analysis reveals increased RelA protein expression and phosphorylation in RWPE-1 cells after TNFα (100ng/mL, 24h) and IFNγ (100ng/mL, 24h) treatment in an additive manner. β-actin is used as a loading control. Representative blot from n=3.
